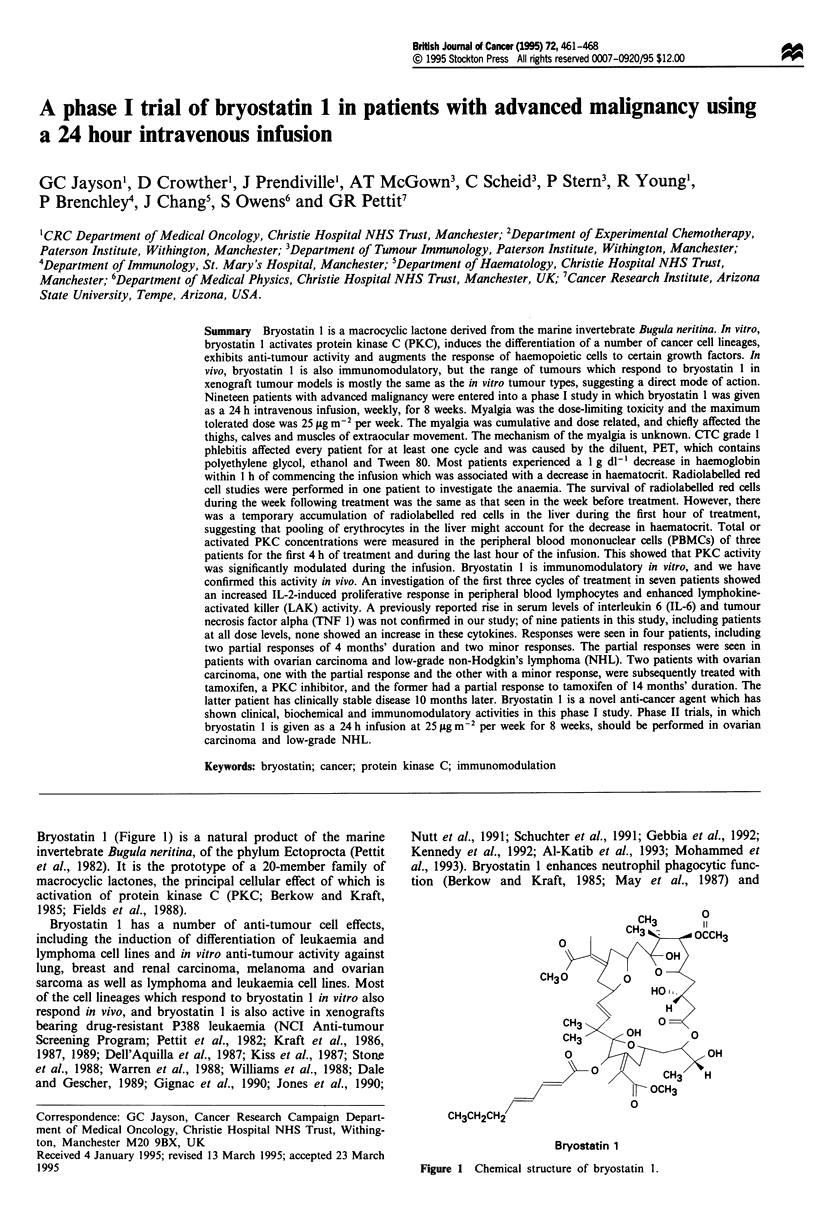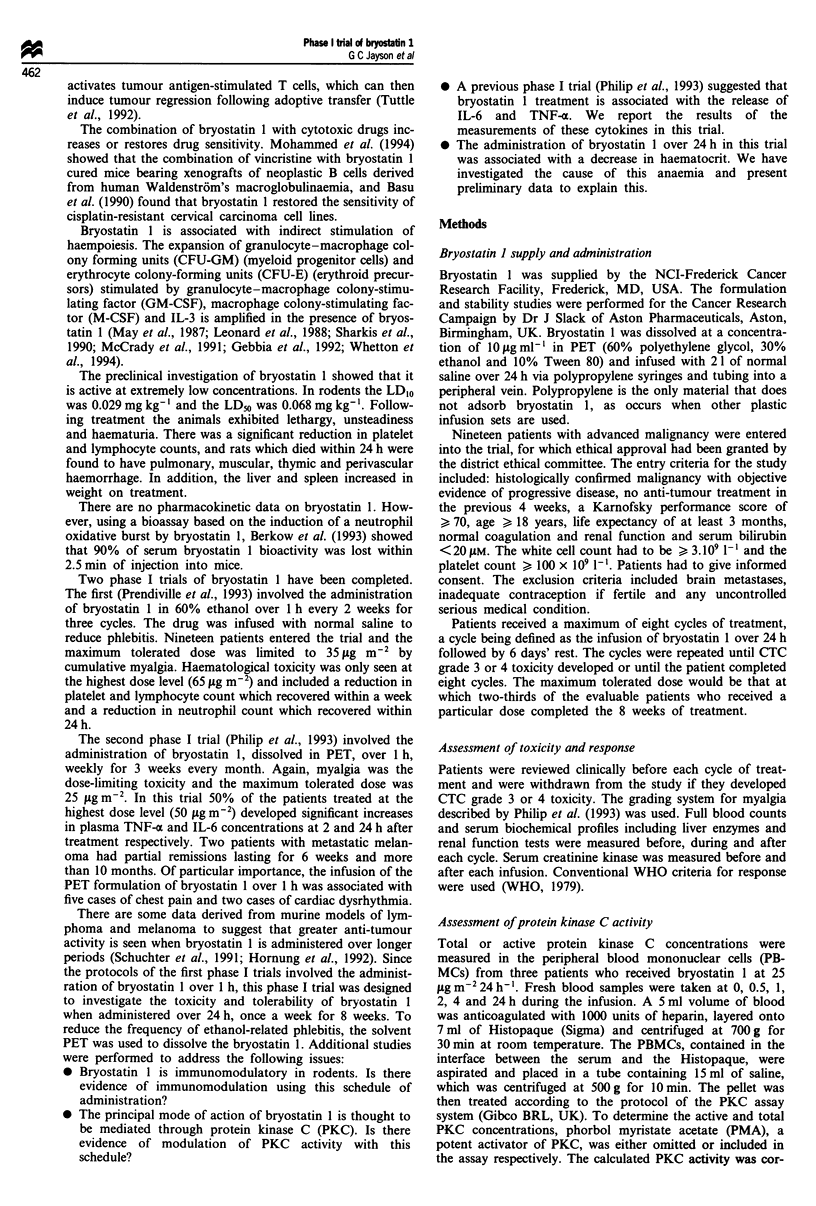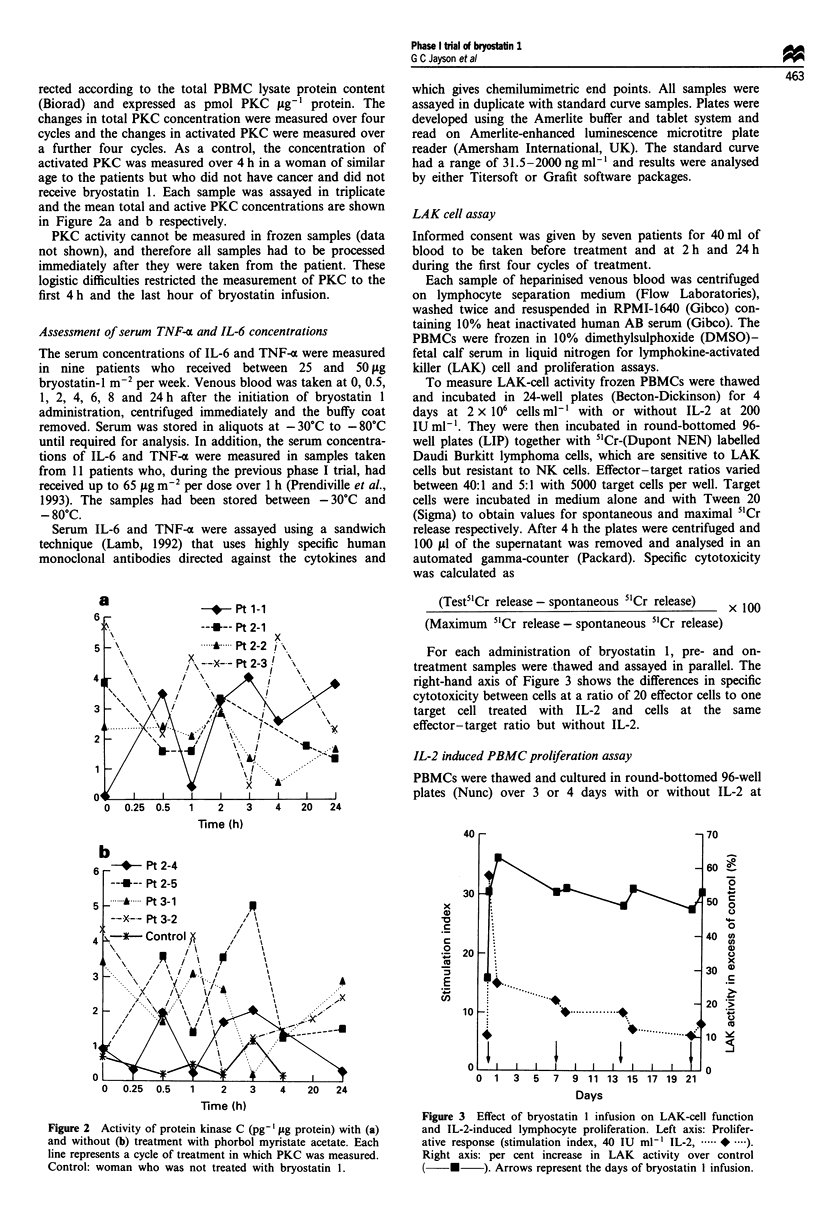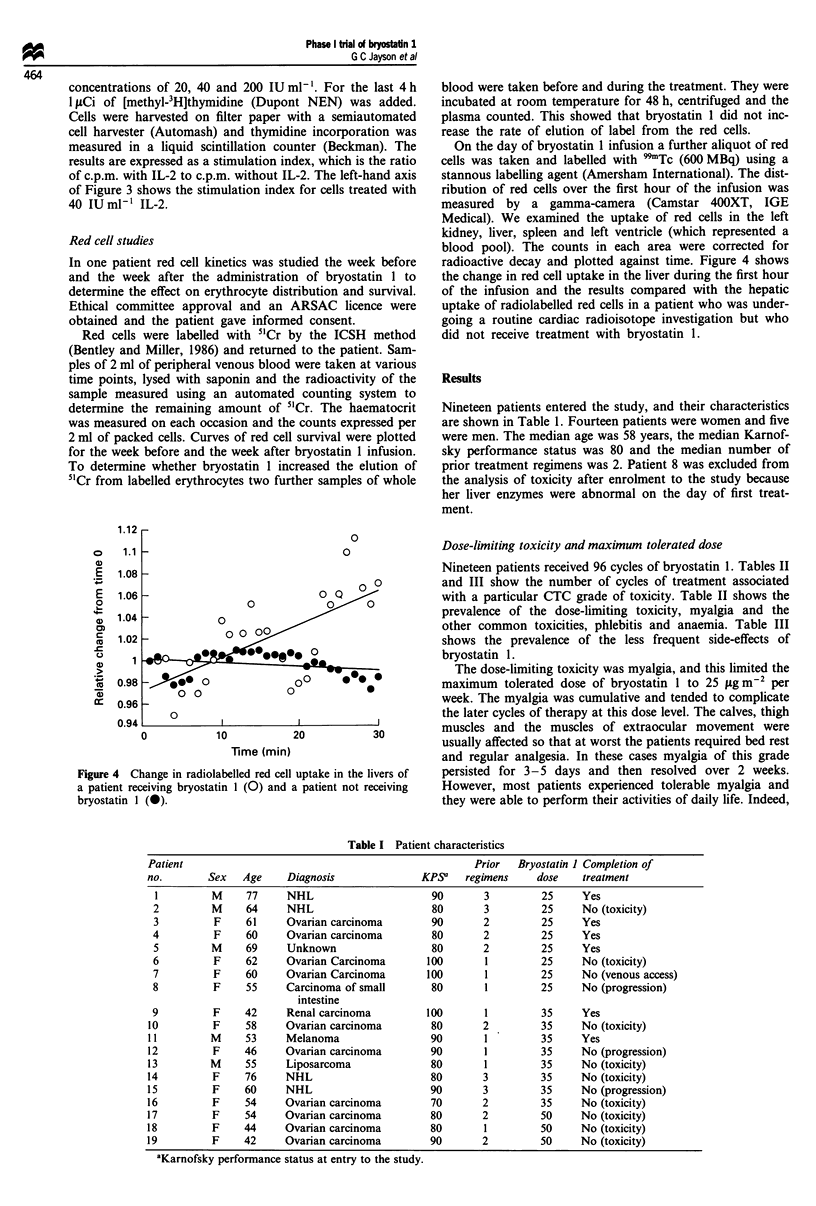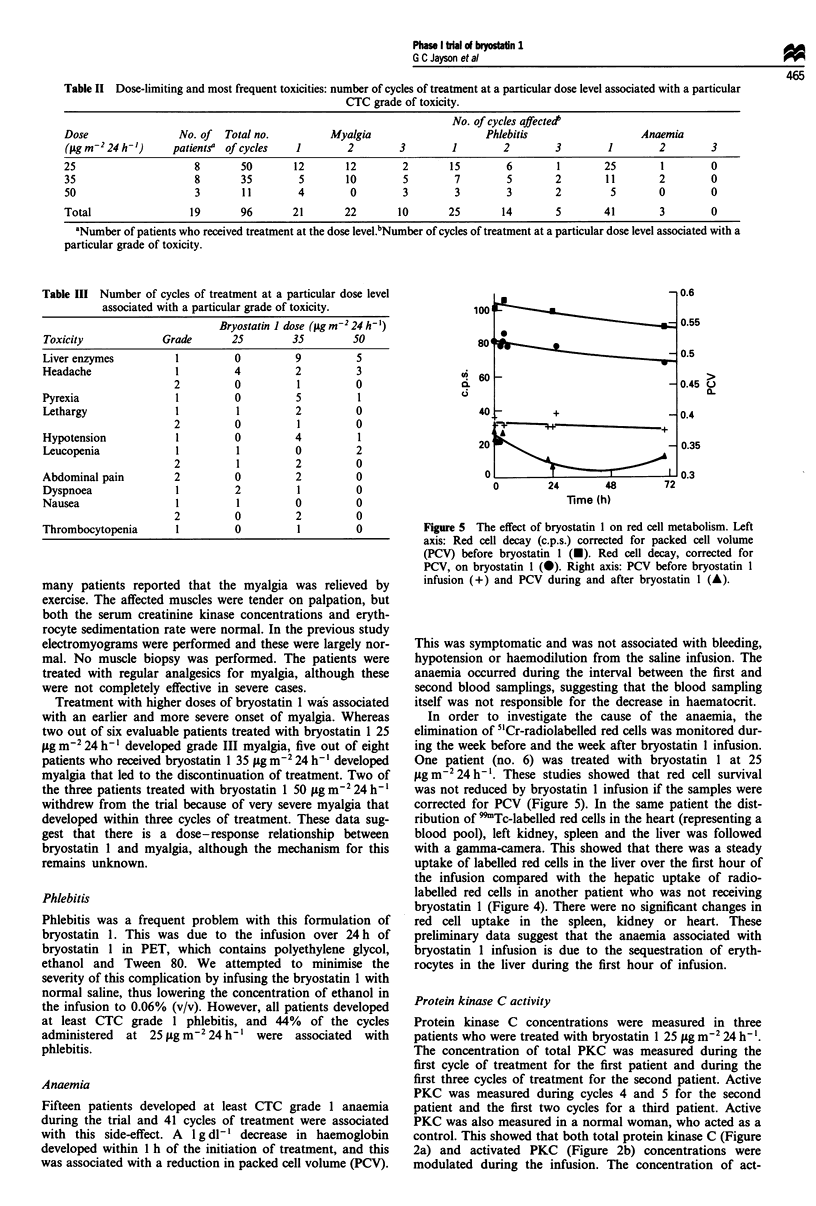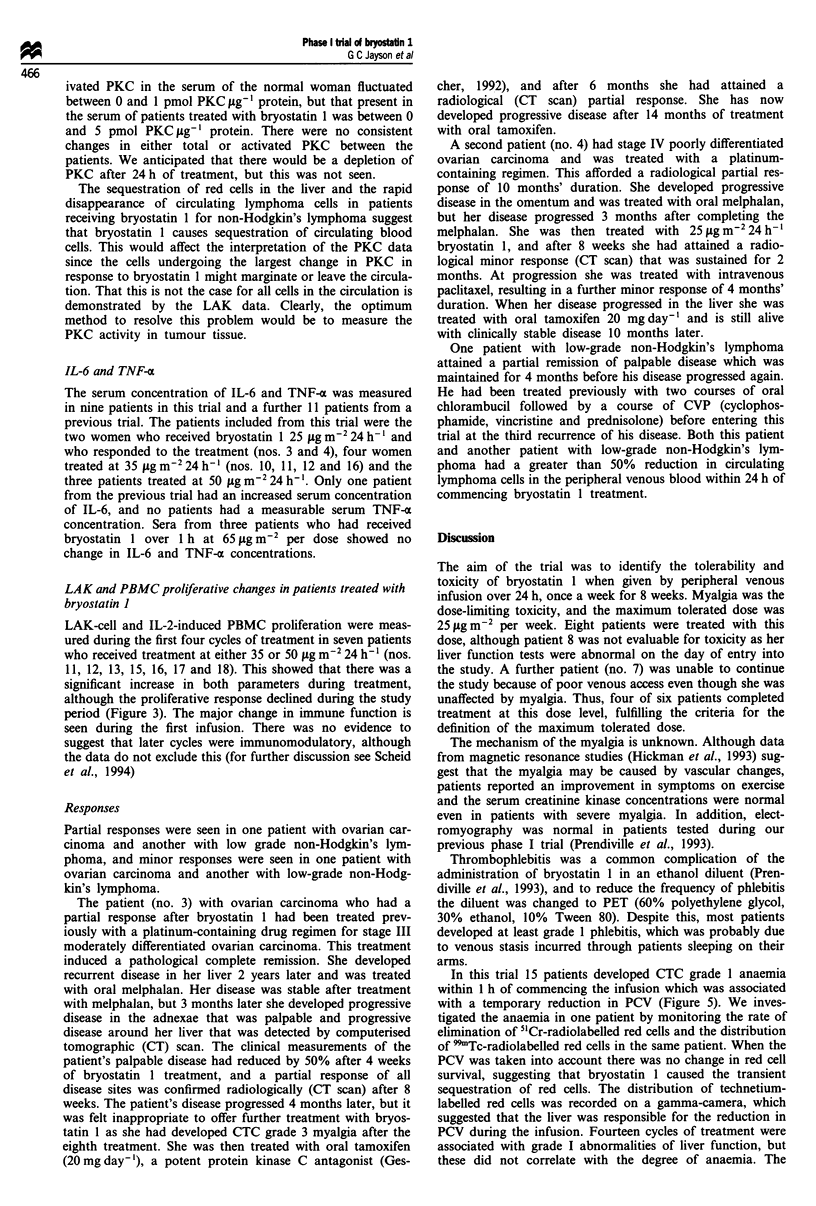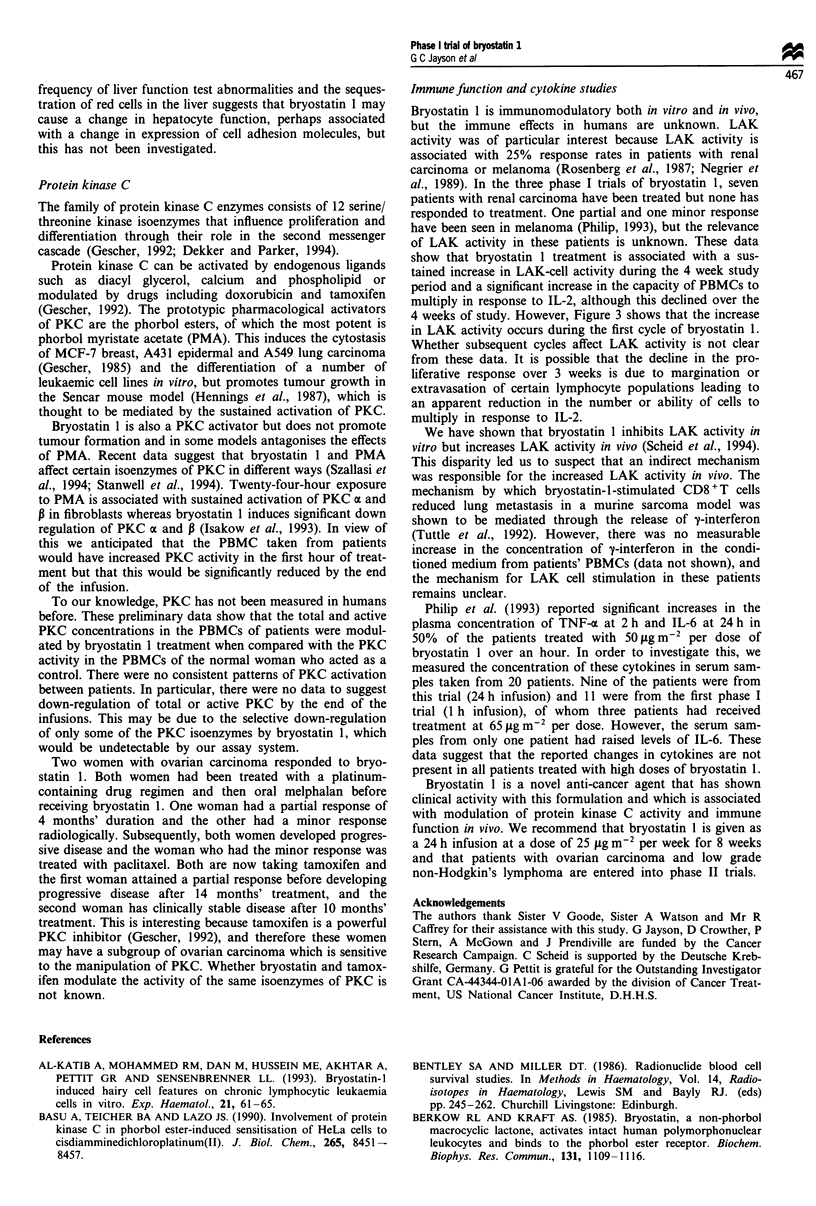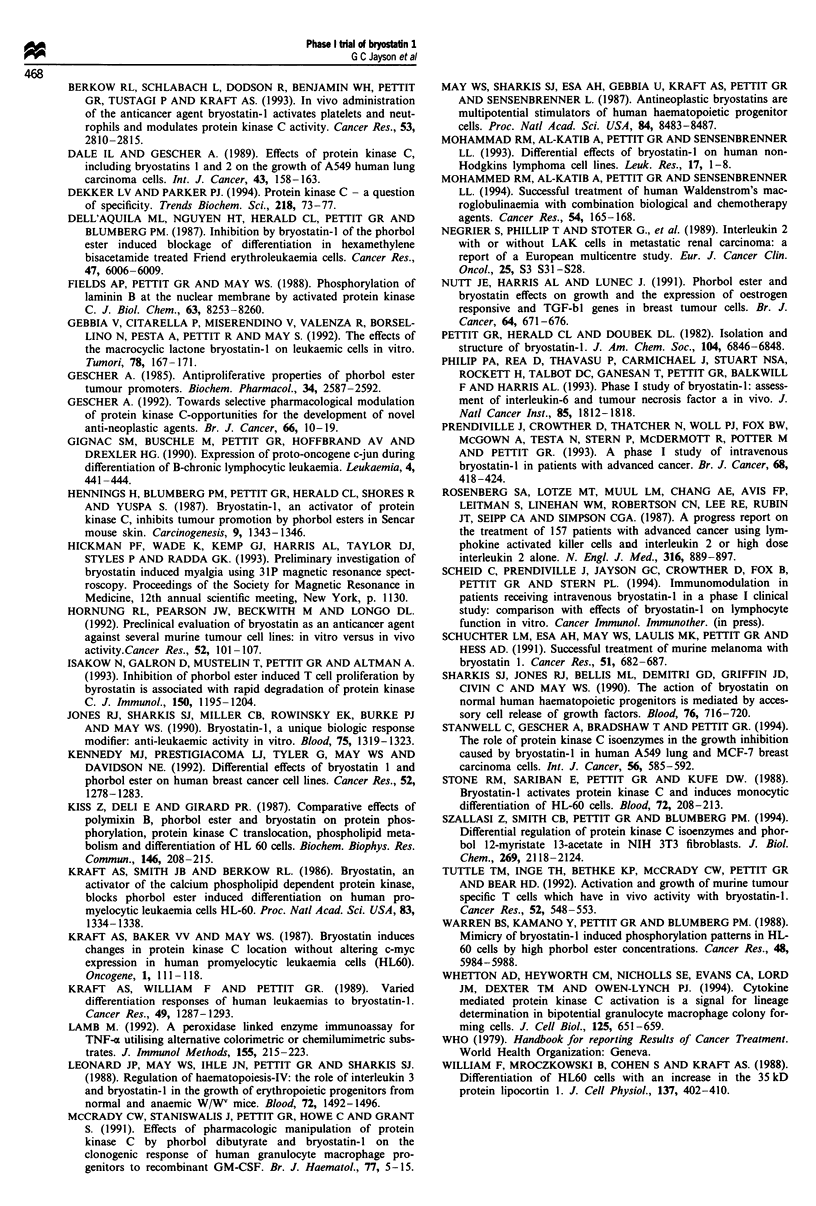# A phase I trial of bryostatin 1 in patients with advanced malignancy using a 24 hour intravenous infusion.

**DOI:** 10.1038/bjc.1995.356

**Published:** 1995-08

**Authors:** G. C. Jayson, D. Crowther, J. Prendiville, A. T. McGown, C. Scheid, P. Stern, R. Young, P. Brenchley, J. Chang, S. Owens

**Affiliations:** CRC Department of Medical Oncology, Christie Hospital NHS Trust, Manchester, UK.

## Abstract

Bryostatin 1 is a macrocyclic lactone derived from the marine invertebrate Bugula neritina. In vitro, bryostatin 1 activates protein kinase C (PKC), induces the differentiation of a number of cancer cell lineages, exhibits anti-tumour activity and augments the response of haemopoietic cells to certain growth factors. In vivo, bryostatin 1 is also immunomodulatory, but the range of tumours which respond to bryostatin 1 in xenograft tumour models is mostly the same as the in vitro tumour types, suggesting a direct mode of action. Nineteen patients with advanced malignancy were entered into a phase I study in which bryostatin 1 was given as a 24 h intravenous infusion, weekly, for 8 weeks. Myalgia was the dose-limiting toxicity and the maximum tolerated dose was 25 micrograms m-2 per week. The myalgia was cumulative and dose related, and chiefly affected the thighs, calves and muscles of extraocular movement. The mechanism of the myalgia is unknown. CTC grade 1 phlebitis affected every patient for at least one cycle and was caused by the diluent, PET, which contains polyethylene glycol, ethanol and Tween 80. Most patients experienced a 1 g dl-1 decrease in haemoglobin within 1 h of commencing the infusion which was associated with a decrease in haematocrit. Radiolabelled red cell studies were performed in one patient to investigate the anaemia. The survival of radiolabelled red cells during the week following treatment was the same as that seen in the week before treatment. However, there was a temporary accumulation of radiolabelled red cells in the liver during the first hour of treatment, suggesting that pooling of erythrocytes in the liver might account for the decrease in haematocrit. Total or activated PKC concentrations were measured in the peripheral blood mononuclear cells (PBMCs) of three patients for the first 4 h of treatment and during the last hour of the infusion. This showed that PKC activity was significantly modulated during the infusion. Bryostatin 1 is immunomodulatory in vitro, and we have confirmed this activity in vivo. An investigation of the first three cycles of treatment in seven patients showed an increased IL-2-induced proliferative response in peripheral blood lymphocytes and enhanced lymphokine-activated killer (LAK) activity. A previously reported rise in serum levels of interleukin 6 (IL-6) and tumour necrosis factor alpha (TNF 1) was not confirmed in our study; of nine patients in this study, including patients at all dose levels, none showed an increase in these cytokines.(ABSTRACT TRUNCATED AT 400 WORDS)


British Journal of Cancer (1995) 72, 461–468
© 1995 Stockton Press All rights reserved 0007-0920/95 $12.00

### A phase I trial of bryostatin 1 in patients with advanced malignancy using a 24 hour intravenous infusion

GC Jayson[1], D Crowther[1], J Prendiville[1], AT McGown[3], C Scheid[3], P Stern[3], R Young[1], P Brenchley[4], J Chang[5], S Owens[6] and GR Pettit[7]

[1]*CRC Department of Medical Oncology, Christie Hospital NHS Trust, Manchester;* [2]*Department of Experimental Chemotherapy, Paterson Institute, Withington, Manchester;* [3]*Department of Tumour Immunology, Paterson Institute, Withington, Manchester;* [4]*Department of Immunology, St. Mary's Hospital, Manchester;* [5]*Department of Haematology, Christie Hospital NHS Trust, Manchester;* [6]*Department of Medical Physics, Christie Hospital NHS Trust, Manchester, UK;* [7]*Cancer Research Institute, Arizona State University, Tempe, Arizona, USA.*

**Summary** Bryostatin 1 is a macrocyclic lactone derived from the marine invertebrate *Bugula neritina*. *In vitro*, bryostatin 1 activates protein kinase C (PKC), induces the differentiation of a number of cancer cell lineages, exhibits anti-tumour activity and augments the response of haemopoietic cells to certain growth factors. *In vivo*, bryostatin 1 is also immunomodulatory, but the range of tumours which respond to bryostatin 1 in xenograft tumour models is mostly the same as the *in vitro* tumour types, suggesting a direct mode of action. Nineteen patients with advanced malignancy were entered into a phase I study in which bryostatin 1 was given as a 24 h intravenous infusion, weekly, for 8 weeks. Myalgia was the dose-limiting toxicity and the maximum tolerated dose was 25 $\mu g\ m^{-2}$ per week. The myalgia was cumulative and dose related, and chiefly affected the thighs, calves and muscles of extraocular movement. The mechanism of the myalgia is unknown. CTC grade 1 phlebitis affected every patient for at least one cycle and was caused by the diluent, PET, which contains polyethylene glycol, ethanol and Tween 80. Most patients experienced a 1 g $dl^{-1}$ decrease in haemoglobin within 1 h of commencing the infusion which was associated with a decrease in haematocrit. Radiolabelled red cell studies were performed in one patient to investigate the anaemia. The survival of radiolabelled red cells during the week following treatment was the same as that seen in the week before treatment. However, there was a temporary accumulation of radiolabelled red cells in the liver during the first hour of treatment, suggesting that pooling of erythrocytes in the liver might account for the decrease in haematocrit. Total or activated PKC concentrations were measured in the peripheral blood mononuclear cells (PBMCs) of three patients for the first 4 h of treatment and during the last hour of the infusion. This showed that PKC activity was significantly modulated during the infusion. Bryostatin 1 is immunomodulatory *in vitro*, and we have confirmed this activity *in vivo*. An investigation of the first three cycles of treatment in seven patients showed an increased IL-2-induced proliferative response in peripheral blood lymphocytes and enhanced lymphokine-activated killer (LAK) activity. A previously reported rise in serum levels of interleukin 6 (IL-6) and tumour necrosis factor alpha (TNF 1) was not confirmed in our study; of nine patients in this study, including patients at all dose levels, none showed an increase in these cytokines. Responses were seen in four patients, including two partial responses of 4 months' duration and two minor responses. The partial responses were seen in patients with ovarian carcinoma and low-grade non-Hodgkin's lymphoma (NHL). Two patients with ovarian carcinoma, one with the partial response and the other with a minor response, were subsequently treated with tamoxifen, a PKC inhibitor, and the former had a partial response to tamoxifen of 14 months' duration. The latter patient has clinically stable disease 10 months later. Bryostatin 1 is a novel anti-cancer agent which has shown clinical, biochemical and immunomodulatory activities in this phase I study. Phase II trials, in which bryostatin 1 is given as a 24 h infusion at 25 $\mu g\ m^{-2}$ per week for 8 weeks, should be performed in ovarian carcinoma and low-grade NHL.

**Keywords:** bryostatin; cancer; protein kinase C; immunomodulation

Bryostatin 1 (Figure 1) is a natural product of the marine invertebrate *Bugula neritina*, of the phylum Ectoprocta (Pettit *et al.*, 1982). It is the prototype of a 20-member family of macrocyclic lactones, the principal cellular effect of which is activation of protein kinase C (PKC; Berkow and Kraft, 1985; Fields *et al.*, 1988).

Bryostatin 1 has a number of anti-tumour cell effects, including the induction of differentiation of leukaemia and lymphoma cell lines and *in vitro* anti-tumour activity against lung, breast and renal carcinoma, melanoma and ovarian sarcoma as well as lymphoma and leukaemia cell lines. Most of the cell lineages which respond to bryostatin 1 *in vitro* also respond *in vivo*, and bryostatin 1 is also active in xenografts bearing drug-resistant P388 leukaemia (NCI Anti-tumour Screening Program; Pettit *et al.*, 1982; Kraft *et al.*, 1986, 1987, 1989; Dell'Aquilla *et al.*, 1987; Kiss *et al.*, 1987; Stone *et al.*, 1988; Warren *et al.*, 1988; Williams *et al.*, 1988; Dale and Gescher, 1989; Gignac *et al.*, 1990; Jones *et al.*, 1990; Nutt *et al.*, 1991; Schuchter *et al.*, 1991; Gebbia *et al.*, 1992; Kennedy *et al.*, 1992; Al-Katib *et al.*, 1993; Mohammed *et al.*, 1993). Bryostatin 1 enhances neutrophil phagocytic function (Berkow and Kraft, 1985; May *et al.*, 1987) and

**Figure 1** Chemical structure of bryostatin 1.

Correspondence: GC Jayson, Cancer Research Campaign Department of Medical Oncology, Christie Hospital NHS Trust, Withington, Manchester M20 9BX, UK
Received 4 January 1995; revised 13 March 1995; accepted 23 March 1995

activates tumour antigen-stimulated T cells, which can then induce tumour regression following adoptive transfer (Tuttle et al., 1992).

The combination of bryostatin 1 with cytotoxic drugs increases or restores drug sensitivity. Mohammed et al. (1994) showed that the combination of vincristine with bryostatin 1 cured mice bearing xenografts of neoplastic B cells derived from human Waldenström's macroglobulinaemia, and Basu et al. (1990) found that bryostatin 1 restored the sensitivity of cisplatin-resistant cervical carcinoma cell lines.

Bryostatin 1 is associated with indirect stimulation of haempoiesis. The expansion of granulocyte–macrophage colony forming units (CFU-GM) (myeloid progenitor cells) and erythrocyte colony-forming units (CFU-E) (erythroid precursors) stimulated by granulocyte–macrophage colony-stimulating factor (GM-CSF), macrophage colony-stimulating factor (M-CSF) and IL-3 is amplified in the presence of bryostatin 1 (May et al., 1987; Leonard et al., 1988; Sharkis et al., 1990; McCrady et al., 1991; Gebbia et al., 1992; Whetton et al., 1994).

The preclinical investigation of bryostatin 1 showed that it is active at extremely low concentrations. In rodents the $LD_{10}$ was 0.029 mg $kg^{-1}$ and the $LD_{50}$ was 0.068 mg $kg^{-1}$. Following treatment the animals exhibited lethargy, unsteadiness and haematuria. There was a significant reduction in platelet and lymphocyte counts, and rats which died within 24 h were found to have pulmonary, muscular, thymic and perivascular haemorrhage. In addition, the liver and spleen increased in weight on treatment.

There are no pharmacokinetic data on bryostatin 1. However, using a bioassay based on the induction of a neutrophil oxidative burst by bryostatin 1, Berkow et al. (1993) showed that 90% of serum bryostatin 1 bioactivity was lost within 2.5 min of injection into mice.

Two phase I trials of bryostatin 1 have been completed. The first (Prendiville et al., 1993) involved the administration of bryostatin 1 in 60% ethanol over 1 h every 2 weeks for three cycles. The drug was infused with normal saline to reduce phlebitis. Nineteen patients entered the trial and the maximum tolerated dose was limited to 35 $\mu g\ m^{-2}$ by cumulative myalgia. Haematological toxicity was only seen at the highest dose level (65 $\mu g\ m^{-2}$) and included a reduction in platelet and lymphocyte count which recovered within a week and a reduction in neutrophil count which recovered within 24 h.

The second phase I trial (Philip et al., 1993) involved the administration of bryostatin 1, dissolved in PET, over 1 h, weekly for 3 weeks every month. Again, myalgia was the dose-limiting toxicity and the maximum tolerated dose was 25 $\mu g\ m^{-2}$. In this trial 50% of the patients treated at the highest dose level (50 $\mu g\ m^{-2}$) developed significant increases in plasma TNF-α and IL-6 concentrations at 2 and 24 h after treatment respectively. Two patients with metastatic melanoma had partial remissions lasting for 6 weeks and more than 10 months. Of particular importance, the infusion of the PET formulation of bryostatin 1 over 1 h was associated with five cases of chest pain and two cases of cardiac dysrhythmia.

There are some data derived from murine models of lymphoma and melanoma to suggest that greater anti-tumour activity is seen when bryostatin 1 is administered over longer periods (Schuchter et al., 1991; Hornung et al., 1992). Since the protocols of the first phase I trials involved the administration of bryostatin 1 over 1 h, this phase I trial was designed to investigate the toxicity and tolerability of bryostatin 1 when administered over 24 h, once a week for 8 weeks. To reduce the frequency of ethanol-related phlebitis, the solvent PET was used to dissolve the bryostatin 1. Additional studies were performed to address the following issues:

- Bryostatin 1 is immunomodulatory in rodents. Is there evidence of immunomodulation using this schedule of administration?
- The principal mode of action of bryostatin 1 is thought to be mediated through protein kinase C (PKC). Is there evidence of modulation of PKC activity with this schedule?
- A previous phase I trial (Philip et al., 1993) suggested that bryostatin 1 treatment is associated with the release of IL-6 and TNF-α. We report the results of the measurements of these cytokines in this trial.
- The administration of bryostatin 1 over 24 h in this trial was associated with a decrease in haematocrit. We have investigated the cause of this anaemia and present preliminary data to explain this.

### Methods

### *Bryostatin 1 supply and administration*

Bryostatin 1 was supplied by the NCI-Frederick Cancer Research Facility, Frederick, MD, USA. The formulation and stability studies were performed for the Cancer Research Campaign by Dr J Slack of Aston Pharmaceuticals, Aston, Birmingham, UK. Bryostatin 1 was dissolved at a concentration of 10 $\mu g\ ml^{-1}$ in PET (60% polyethylene glycol, 30% ethanol and 10% Tween 80) and infused with 2 l of normal saline over 24 h via polypropylene syringes and tubing into a peripheral vein. Polypropylene is the only material that does not adsorb bryostatin 1, as occurs when other plastic infusion sets are used.

Nineteen patients with advanced malignancy were entered into the trial, for which ethical approval had been granted by the district ethical committee. The entry criteria for the study included: histologically confirmed malignancy with objective evidence of progressive disease, no anti-tumour treatment in the previous 4 weeks, a Karnofsky performance score of $\geqslant 70$, age $\geqslant 18$ years, life expectancy of at least 3 months, normal coagulation and renal function and serum bilirubin $<20\ \mu M$. The white cell count had to be $\geqslant 3.10^9\ l^{-1}$ and the platelet count $\geqslant 100 \times 10^9\ l^{-1}$. Patients had to give informed consent. The exclusion criteria included brain metastases, inadequate contraception if fertile and any uncontrolled serious medical condition.

Patients received a maximum of eight cycles of treatment, a cycle being defined as the infusion of bryostatin 1 over 24 h followed by 6 days' rest. The cycles were repeated until CTC grade 3 or 4 toxicity developed or until the patient completed eight cycles. The maximum tolerated dose would be that at which two-thirds of the evaluable patients who received a particular dose completed the 8 weeks of treatment.

### *Assessment of toxicity and response*

Patients were reviewed clinically before each cycle of treatment and were withdrawn from the study if they developed CTC grade 3 or 4 toxicity. The grading system for myalgia described by Philip et al. (1993) was used. Full blood counts and serum biochemical profiles including liver enzymes and renal function tests were measured before, during and after each cycle. Serum creatinine kinase was measured before and after each infusion. Conventional WHO criteria for response were used (WHO, 1979).

### *Assessment of protein kinase C activity*

Total or active protein kinase C concentrations were measured in the peripheral blood mononuclear cells (PBMCs) from three patients who received bryostatin 1 at 25 $\mu g\ m^{-2}\ 24\ h^{-1}$. Fresh blood samples were taken at 0, 0.5, 1, 2, 4 and 24 h during the infusion. A 5 ml volume of blood was anticoagulated with 1000 units of heparin, layered onto 7 ml of Histopaque (Sigma) and centrifuged at 700 g for 30 min at room temperature. The PBMCs, contained in the interface between the serum and the Histopaque, were aspirated and placed in a tube containing 15 ml of saline, which was centrifuged at 500 g for 10 min. The pellet was then treated according to the protocol of the PKC assay system (Gibco BRL, UK). To determine the active and total PKC concentrations, phorbol myristate acetate (PMA), a potent activator of PKC, was either omitted or included in the assay respectively. The calculated PKC activity was corrected according to the total PBMC lysate protein content (Biorad) and expressed as pmol PKC $\mu g^{-1}$ protein. The changes in total PKC concentration were measured over four cycles and the changes in activated PKC were measured over a further four cycles. As a control, the concentration of activated PKC was measured over 4 h in a woman of similar age to the patients but who did not have cancer and did not receive bryostatin 1. Each sample was assayed in triplicate and the mean total and active PKC concentrations are shown in Figure 2a and b respectively.

PKC activity cannot be measured in frozen samples (data not shown), and therefore all samples had to be processed immediately after they were taken from the patient. These logistic difficulties restricted the measurement of PKC to the first 4 h and the last hour of bryostatin infusion.

*Assessment of serum TNF-α and IL-6 concentrations*

The serum concentrations of IL-6 and TNF-α were measured in nine patients who received between 25 and 50 µg bryostatin-1 $m^{-2}$ per week. Venous blood was taken at 0, 0.5, 1, 2, 4, 6, 8 and 24 h after the initiation of bryostatin 1 administration, centrifuged immediately and the buffy coat removed. Serum was stored in aliquots at −30°C to −80°C until required for analysis. In addition, the serum concentrations of IL-6 and TNF-α were measured in samples taken from 11 patients who, during the previous phase I trial, had received up to 65 µg $m^{-2}$ per dose over 1 h (Prendiville *et al.*, 1993). The samples had been stored between −30°C and −80°C.

Serum IL-6 and TNF-α were assayed using a sandwich technique (Lamb, 1992) that uses highly specific human monoclonal antibodies directed against the cytokines and which gives chemilumimetric end points. All samples were assayed in duplicate with standard curve samples. Plates were developed using the Amerlite buffer and tablet system and read on Amerlite-enhanced luminescence microtitre plate reader (Amersham International, UK). The standard curve had a range of 31.5–2000 ng $ml^{-1}$ and results were analysed by either Titersoft or Grafit software packages.

*LAK cell assay*

Informed consent was given by seven patients for 40 ml of blood to be taken before treatment and at 2 h and 24 h during the first four cycles of treatment.

Each sample of heparinised venous blood was centrifuged on lymphocyte separation medium (Flow Laboratories), washed twice and resuspended in RPMI-1640 (Gibco) containing 10% heat inactivated human AB serum (Gibco). The PBMCs were frozen in 10% dimethylsulphoxide (DMSO)–fetal calf serum in liquid nitrogen for lymphokine-activated killer (LAK) cell and proliferation assays.

To measure LAK-cell activity frozen PBMCs were thawed and incubated in 24-well plates (Becton-Dickinson) for 4 days at $2 \times 10^6$ cells $ml^{-1}$ with or without IL-2 at 200 IU $ml^{-1}$. They were then incubated in round-bottomed 96-well plates (LIP) together with $^{51}Cr$-(Dupont NEN) labelled Daudi Burkitt lymphoma cells, which are sensitive to LAK cells but resistant to NK cells. Effector–target ratios varied between 40:1 and 5:1 with 5000 target cells per well. Target cells were incubated in medium alone and with Tween 20 (Sigma) to obtain values for spontaneous and maximal $^{51}Cr$ release respectively. After 4 h the plates were centrifuged and 100 µl of the supernatant was removed and analysed in an automated gamma-counter (Packard). Specific cytotoxicity was calculated as

$$\frac{(\text{Test}^{51}\text{Cr release} - \text{spontaneous }^{51}\text{Cr release})}{(\text{Maximum }^{51}\text{Cr release} - \text{spontaneous }^{51}\text{Cr release})} \times 100$$

For each administration of bryostatin 1, pre- and on-treatment samples were thawed and assayed in parallel. The right-hand axis of Figure 3 shows the differences in specific cytotoxicity between cells at a ratio of 20 effector cells to one target cell treated with IL-2 and cells at the same effector–target ratio but without IL-2.

*IL-2 induced PBMC proliferation assay*

PBMCs were thawed and cultured in round-bottomed 96-well plates (Nunc) over 3 or 4 days with or without IL-2 at

**Figure 2** Activity of protein kinase C ($pg^{-1}$ µg protein) with (**a**) and without (**b**) treatment with phorbol myristate acetate. Each line represents a cycle of treatment in which PKC was measured. Control: woman who was not treated with bryostatin 1.

**Figure 3** Effect of bryostatin 1 infusion on LAK-cell function and IL-2-induced lymphocyte proliferation. Left axis: Proliferative response (stimulation index, 40 IU $ml^{-1}$ IL-2, ····◆····). Right axis: per cent increase in LAK activity over control (——■——). Arrows represent the days of bryostatin 1 infusion.

concentrations of 20, 40 and 200 IU ml$^{-1}$. For the last 4 h 1 $\mu$Ci of [methyl-$^3$H]thymidine (Dupont NEN) was added. Cells were harvested on filter paper with a semiautomated cell harvester (Automash) and thymidine incorporation was measured in a liquid scintillation counter (Beckman). The results are expressed as a stimulation index, which is the ratio of c.p.m. with IL-2 to c.p.m. without IL-2. The left-hand axis of Figure 3 shows the stimulation index for cells treated with 40 IU ml$^{-1}$ IL-2.

*Red cell studies*

In one patient red cell kinetics was studied the week before and the week after the administration of bryostatin 1 to determine the effect on erythrocyte distribution and survival. Ethical committee approval and an ARSAC licence were obtained and the patient gave informed consent.

Red cells were labelled with $^{51}$Cr by the ICSH method (Bentley and Miller, 1986) and returned to the patient. Samples of 2 ml of peripheral venous blood were taken at various time points, lysed with saponin and the radioactivity of the sample measured using an automated counting system to determine the remaining amount of $^{51}$Cr. The haematocrit was measured on each occasion and the counts expressed per 2 ml of packed cells. Curves of red cell survival were plotted for the week before and the week after bryostatin 1 infusion. To determine whether bryostatin 1 increased the elution of $^{51}$Cr from labelled erythrocytes two further samples of whole blood were taken before and during the treatment. They were incubated at room temperature for 48 h, centrifuged and the plasma counted. This showed that bryostatin 1 did not increase the rate of elution of label from the red cells.

On the day of bryostatin 1 infusion a further aliquot of red cells was taken and labelled with $^{99m}$Tc (600 MBq) using a stannous labelling agent (Amersham International). The distribution of red cells over the first hour of the infusion was measured by a gamma-camera (Camstar 400XT, IGE Medical). We examined the uptake of red cells in the left kidney, liver, spleen and left ventricle (which represented a blood pool). The counts in each area were corrected for radioactive decay and plotted against time. Figure 4 shows the change in red cell uptake in the liver during the first hour of the infusion and the results compared with the hepatic uptake of radiolabelled red cells in a patient who was undergoing a routine cardiac radioisotope investigation but who did not receive treatment with bryostatin 1.

**Figure 4** Change in radiolabelled red cell uptake in the livers of a patient receiving bryostatin 1 (○) and a patient not receiving bryostatin 1 (●).

### Results

Nineteen patients entered the study, and their characteristics are shown in Table 1. Fourteen patients were women and five were men. The median age was 58 years, the median Karnofsky performance status was 80 and the median number of prior treatment regimens was 2. Patient 8 was excluded from the analysis of toxicity after enrolment to the study because her liver enzymes were abnormal on the day of first treatment.

*Dose-limiting toxicity and maximum tolerated dose*

Nineteen patients received 96 cycles of bryostatin 1. Tables II and III show the number of cycles of treatment associated with a particular CTC grade of toxicity. Table II shows the prevalence of the dose-limiting toxicity, myalgia and the other common toxicities, phlebitis and anaemia. Table III shows the prevalence of the less frequent side-effects of bryostatin 1.

The dose-limiting toxicity was myalgia, and this limited the maximum tolerated dose of bryostatin 1 to 25 $\mu$g m$^{-2}$ per week. The myalgia was cumulative and tended to complicate the later cycles of therapy at this dose level. The calves, thigh muscles and the muscles of extraocular movement were usually affected so that at worst the patients required bed rest and regular analgesia. In these cases myalgia of this grade persisted for 3–5 days and then resolved over 2 weeks. However, most patients experienced tolerable myalgia and they were able to perform their activities of daily life. Indeed,

**Table I** Patient characteristics

| *Patient no.* | *Sex* | *Age* | *Diagnosis* | *KPS*[a] | *Prior regimens* | *Bryostatin 1 dose* | *Completion of treatment* |
|---|---|---|---|---|---|---|---|
| 1 | M | 77 | NHL | 90 | 3 | 25 | Yes |
| 2 | M | 64 | NHL | 80 | 3 | 25 | No (toxicity) |
| 3 | F | 61 | Ovarian carcinoma | 90 | 2 | 25 | Yes |
| 4 | F | 60 | Ovarian carcinoma | 80 | 2 | 25 | Yes |
| 5 | M | 69 | Unknown | 80 | 2 | 25 | Yes |
| 6 | F | 62 | Ovarian Carcinoma | 100 | 1 | 25 | No (toxicity) |
| 7 | F | 60 | Ovarian Carcinoma | 100 | 1 | 25 | No (venous access) |
| 8 | F | 55 | Carcinoma of small intestine | 80 | 1 | 25 | No (progression) |
| 9 | F | 42 | Renal carcinoma | 100 | 1 | 35 | Yes |
| 10 | F | 58 | Ovarian carcinoma | 80 | 2 | 35 | No (toxicity) |
| 11 | M | 53 | Melanoma | 90 | 1 | 35 | Yes |
| 12 | F | 46 | Ovarian carcinoma | 90 | 1 | 35 | No (progression) |
| 13 | M | 55 | Liposarcoma | 80 | 1 | 35 | No (toxicity) |
| 14 | F | 76 | NHL | 80 | 3 | 35 | No (toxicity) |
| 15 | F | 60 | NHL | 90 | 3 | 35 | No (progression) |
| 16 | F | 54 | Ovarian carcinoma | 70 | 2 | 35 | No (toxicity) |
| 17 | F | 54 | Ovarian carcinoma | 80 | 2 | 50 | No (toxicity) |
| 18 | F | 44 | Ovarian carcinoma | 80 | 1 | 50 | No (toxicity) |
| 19 | F | 42 | Ovarian carcinoma | 90 | 2 | 50 | No (toxicity) |

[a]Karnofsky performance status at entry to the study.

**Table II** Dose-limiting and most frequent toxicities: number of cycles of treatment at a particular dose level associated with a particular CTC grade of toxicity.

| Dose ($\mu g\ m^{-2}\ 24\ h^{-1}$) | No. of patients[a] | Total no. of cycles | Myalgia 1 | Myalgia 2 | Myalgia 3 | Phlebitis 1 | Phlebitis 2 | Phlebitis 3 | Anaemia 1 | Anaemia 2 | Anaemia 3 |
|---|---|---|---|---|---|---|---|---|---|---|---|
| | | | *No. of cycles affected*[b] | | | | | | | | |
| 25 | 8 | 50 | 12 | 12 | 2 | 15 | 6 | 1 | 25 | 1 | 0 |
| 35 | 8 | 35 | 5 | 10 | 5 | 7 | 5 | 2 | 11 | 2 | 0 |
| 50 | 3 | 11 | 4 | 0 | 3 | 3 | 3 | 2 | 5 | 0 | 0 |
| Total | 19 | 96 | 21 | 22 | 10 | 25 | 14 | 5 | 41 | 3 | 0 |

[a]Number of patients who received treatment at the dose level.[b]Number of cycles of treatment at a particular dose level associated with a particular grade of toxicity.

**Table III** Number of cycles of treatment at a particular dose level associated with a particular grade of toxicity.

| Toxicity | Grade | Bryostatin 1 dose ($\mu g\ m^{-2}\ 24\ h^{-1}$) 25 | 35 | 50 |
|---|---|---|---|---|
| Liver enzymes | 1 | 0 | 9 | 5 |
| Headache | 1 | 4 | 2 | 3 |
| | 2 | 0 | 1 | 0 |
| Pyrexia | 1 | 0 | 5 | 1 |
| Lethargy | 1 | 1 | 2 | 0 |
| | 2 | 0 | 1 | 0 |
| Hypotension | 1 | 0 | 4 | 1 |
| Leucopenia | 1 | 1 | 0 | 2 |
| | 2 | 1 | 2 | 0 |
| Abdominal pain | 2 | 0 | 2 | 0 |
| Dyspnoea | 1 | 2 | 1 | 0 |
| Nausea | 1 | 1 | 0 | 0 |
| | 2 | 0 | 2 | 0 |
| Thrombocytopenia | 1 | 0 | 1 | 0 |

many patients reported that the myalgia was relieved by exercise. The affected muscles were tender on palpation, but both the serum creatinine kinase concentrations and erythrocyte sedimentation rate were normal. In the previous study electromyograms were performed and these were largely normal. No muscle biopsy was performed. The patients were treated with regular analgesics for myalgia, although these were not completely effective in severe cases.

Treatment with higher doses of bryostatin 1 was associated with an earlier and more severe onset of myalgia. Whereas two out of six evaluable patients treated with bryostatin 1 25 $\mu g\ m^{-2}\ 24\ h^{-1}$ developed grade III myalgia, five out of eight patients who received bryostatin 1 35 $\mu g\ m^{-2}\ 24\ h^{-1}$ developed myalgia that led to the discontinuation of treatment. Two of the three patients treated with bryostatin 1 50 $\mu g\ m^{-2}\ 24\ h^{-1}$ withdrew from the trial because of very severe myalgia that developed within three cycles of treatment. These data suggest that there is a dose–response relationship between bryostatin 1 and myalgia, although the mechanism for this remains unknown.

*Phlebitis*

Phlebitis was a frequent problem with this formulation of bryostatin 1. This was due to the infusion over 24 h of bryostatin 1 in PET, which contains polyethylene glycol, ethanol and Tween 80. We attempted to minimise the severity of this complication by infusing the bryostatin 1 with normal saline, thus lowering the concentration of ethanol in the infusion to 0.06% (v/v). However, all patients developed at least CTC grade 1 phlebitis, and 44% of the cycles administered at 25 $\mu g\ m^{-2}\ 24\ h^{-1}$ were associated with phlebitis.

*Anaemia*

Fifteen patients developed at least CTC grade 1 anaemia during the trial and 41 cycles of treatment were associated with this side-effect. A 1 g $dl^{-1}$ decrease in haemoglobin developed within 1 h of the initiation of treatment, and this was associated with a reduction in packed cell volume (PCV).

**Figure 5** The effect of bryostatin 1 on red cell metabolism. Left axis: Red cell decay (c.p.s.) corrected for packed cell volume (PCV) before bryostatin 1 (■). Red cell decay, corrected for PCV, on bryostatin 1 (●). Right axis: PCV before bryostatin 1 infusion (+) and PCV during and after bryostatin 1 (▲).

This was symptomatic and was not associated with bleeding, hypotension or haemodilution from the saline infusion. The anaemia occurred during the interval between the first and second blood samplings, suggesting that the blood sampling itself was not responsible for the decrease in haematocrit.

In order to investigate the cause of the anaemia, the elimination of $^{51}Cr$-radiolabelled red cells was monitored during the week before and the week after bryostatin 1 infusion. One patient (no. 6) was treated with bryostatin 1 at 25 $\mu g\ m^{-2}\ 24\ h^{-1}$. These studies showed that red cell survival was not reduced by bryostatin 1 infusion if the samples were corrected for PCV (Figure 5). In the same patient the distribution of $^{99m}Tc$-labelled red cells in the heart (representing a blood pool), left kidney, spleen and the liver was followed with a gamma-camera. This showed that there was a steady uptake of labelled red cells in the liver over the first hour of the infusion compared with the hepatic uptake of radiolabelled red cells in another patient who was not receiving bryostatin 1 (Figure 4). There were no significant changes in red cell uptake in the spleen, kidney or heart. These preliminary data suggest that the anaemia associated with bryostatin 1 infusion is due to the sequestration of erythrocytes in the liver during the first hour of infusion.

*Protein kinase C activity*

Protein kinase C concentrations were measured in three patients who were treated with bryostatin 1 25 $\mu g\ m^{-2}\ 24\ h^{-1}$. The concentration of total PKC was measured during the first cycle of treatment for the first patient and during the first three cycles of treatment for the second patient. Active PKC was measured during cycles 4 and 5 for the second patient and the first two cycles for a third patient. Active PKC was also measured in a normal woman, who acted as a control. This showed that both total protein kinase C (Figure 2a) and activated PKC (Figure 2b) concentrations were modulated during the infusion. The concentration of activated PKC in the serum of the normal woman fluctuated between 0 and 1 pmol PKC $\mu g^{-1}$ protein, but that present in the serum of patients treated with bryostatin 1 was between 0 and 5 pmol PKC $\mu g^{-1}$ protein. There were no consistent changes in either total or activated PKC between the patients. We anticipated that there would be a depletion of PKC after 24 h of treatment, but this was not seen.

The sequestration of red cells in the liver and the rapid disappearance of circulating lymphoma cells in patients receiving bryostatin 1 for non-Hodgkin's lymphoma suggest that bryostatin 1 causes sequestration of circulating blood cells. This would affect the interpretation of the PKC data since the cells undergoing the largest change in PKC in response to bryostatin 1 might marginate or leave the circulation. That this is not the case for all cells in the circulation is demonstrated by the LAK data. Clearly, the optimum method to resolve this problem would be to measure the PKC activity in tumour tissue.

### *IL-6 and TNF-α*

The serum concentration of IL-6 and TNF-α was measured in nine patients in this trial and a further 11 patients from a previous trial. The patients included from this trial were the two women who received bryostatin 1 25 $\mu g\ m^{-2}\ 24\ h^{-1}$ and who responded to the treatment (nos. 3 and 4), four women treated at 35 $\mu g\ m^{-2}\ 24\ h^{-1}$ (nos. 10, 11, 12 and 16) and the three patients treated at 50 $\mu g\ m^{-2}\ 24\ h^{-1}$. Only one patient from the previous trial had an increased serum concentration of IL-6, and no patients had a measurable serum TNF-α concentration. Sera from three patients who had received bryostatin 1 over 1 h at 65 $\mu g\ m^{-2}$ per dose showed no change in IL-6 and TNF-α concentrations.

### *LAK and PBMC proliferative changes in patients treated with bryostatin 1*

LAK-cell and IL-2-induced PBMC proliferation were measured during the first four cycles of treatment in seven patients who received treatment at either 35 or 50 $\mu g\ m^{-2}\ 24\ h^{-1}$ (nos. 11, 12, 13, 15, 16, 17 and 18). This showed that there was a significant increase in both parameters during treatment, although the proliferative response declined during the study period (Figure 3). The major change in immune function is seen during the first infusion. There was no evidence to suggest that later cycles were immunomodulatory, although the data do not exclude this (for further discussion see Scheid *et al*., 1994)

### *Responses*

Partial responses were seen in one patient with ovarian carcinoma and another with low grade non-Hodgkin's lymphoma, and minor responses were seen in one patient with ovarian carcinoma and another with low-grade non-Hodgkin's lymphoma.

The patient (no. 3) with ovarian carcinoma who had a partial response after bryostatin 1 had been treated previously with a platinum-containing drug regimen for stage III moderately differentiated ovarian carcinoma. This treatment induced a pathological complete remission. She developed recurrent disease in her liver 2 years later and was treated with oral melphalan. Her disease was stable after treatment with melphalan, but 3 months later she developed progressive disease in the adnexae that was palpable and progressive disease around her liver that was detected by computerised tomographic (CT) scan. The clinical measurements of the patient's palpable disease had reduced by 50% after 4 weeks of bryostatin 1 treatment, and a partial response of all disease sites was confirmed radiologically (CT scan) after 8 weeks. The patient's disease progressed 4 months later, but it was felt inappropriate to offer further treatment with bryostatin 1 as she had developed CTC grade 3 myalgia after the eighth treatment. She was then treated with oral tamoxifen (20 mg $day^{-1}$), a potent protein kinase C antagonist (Gescher, 1992), and after 6 months she had attained a radiological (CT scan) partial response. She has now developed progressive disease after 14 months of treatment with oral tamoxifen.

A second patient (no. 4) had stage IV poorly differentiated ovarian carcinoma and was treated with a platinum-containing regimen. This afforded a radiological partial response of 10 months' duration. She developed progressive disease in the omentum and was treated with oral melphalan, but her disease progressed 3 months after completing the melphalan. She was then treated with 25 $\mu g\ m^{-2}\ 24\ h^{-1}$ bryostatin 1, and after 8 weeks she had attained a radiological minor response (CT scan) that was sustained for 2 months. At progression she was treated with intravenous paclitaxel, resulting in a further minor response of 4 months' duration. When her disease progressed in the liver she was treated with oral tamoxifen 20 mg $day^{-1}$ and is still alive with clinically stable disease 10 months later.

One patient with low-grade non-Hodgkin's lymphoma attained a partial remission of palpable disease which was maintained for 4 months before his disease progressed again. He had been treated previously with two courses of oral chlorambucil followed by a course of CVP (cyclophosphamide, vincristine and prednisolone) before entering this trial at the third recurrence of his disease. Both this patient and another patient with low-grade non-Hodgkin's lymphoma had a greater than 50% reduction in circulating lymphoma cells in the peripheral venous blood within 24 h of commencing bryostatin 1 treatment.

### Discussion

The aim of the trial was to identify the tolerability and toxicity of bryostatin 1 when given by peripheral venous infusion over 24 h, once a week for 8 weeks. Myalgia was the dose-limiting toxicity, and the maximum tolerated dose was 25 $\mu g\ m^{-2}$ per week. Eight patients were treated with this dose, although patient 8 was not evaluable for toxicity as her liver function tests were abnormal on the day of entry into the study. A further patient (no. 7) was unable to continue the study because of poor venous access even though she was unaffected by myalgia. Thus, four of six patients completed treatment at this dose level, fulfilling the criteria for the definition of the maximum tolerated dose.

The mechanism of the myalgia is unknown. Although data from magnetic resonance studies (Hickman *et al*., 1993) suggest that the myalgia may be caused by vascular changes, patients reported an improvement in symptoms on exercise and the serum creatinine kinase concentrations were normal even in patients with severe myalgia. In addition, electromyography was normal in patients tested during our previous phase I trial (Prendiville *et al*., 1993).

Thrombophlebitis was a common complication of the administration of bryostatin 1 in an ethanol diluent (Prendiville *et al*., 1993), and to reduce the frequency of phlebitis the diluent was changed to PET (60% polyethylene glycol, 30% ethanol, 10% Tween 80). Despite this, most patients developed at least grade 1 phlebitis, which was probably due to venous stasis incurred through patients sleeping on their arms.

In this trial 15 patients developed CTC grade 1 anaemia within 1 h of commencing the infusion which was associated with a temporary reduction in PCV (Figure 5). We investigated the anaemia in one patient by monitoring the rate of elimination of $^{51}Cr$-radiolabelled red cells and the distribution of $^{99m}Tc$-radiolabelled red cells in the same patient. When the PCV was taken into account there was no change in red cell survival, suggesting that bryostatin 1 caused the transient sequestration of red cells. The distribution of technetium-labelled red cells was recorded on a gamma-camera, which suggested that the liver was responsible for the reduction in PCV during the infusion. Fourteen cycles of treatment were associated with grade I abnormalities of liver function, but these did not correlate with the degree of anaemia. The frequency of liver function test abnormalities and the sequestration of red cells in the liver suggests that bryostatin 1 may cause a change in hepatocyte function, perhaps associated with a change in expression of cell adhesion molecules, but this has not been investigated.

*Protein kinase C*

The family of protein kinase C enzymes consists of 12 serine/threonine kinase isoenzymes that influence proliferation and differentiation through their role in the second messenger cascade (Gescher, 1992; Dekker and Parker, 1994).

Protein kinase C can be activated by endogenous ligands such as diacyl glycerol, calcium and phospholipid or modulated by drugs including doxorubicin and tamoxifen (Gescher, 1992). The prototypic pharmacological activators of PKC are the phorbol esters, of which the most potent is phorbol myristate acetate (PMA). This induces the cytostasis of MCF-7 breast, A431 epidermal and A549 lung carcinoma (Gescher, 1985) and the differentiation of a number of leukaemic cell lines *in vitro*, but promotes tumour growth in the Sencar mouse model (Hennings *et al.*, 1987), which is thought to be mediated by the sustained activation of PKC.

Bryostatin 1 is also a PKC activator but does not promote tumour formation and in some models antagonises the effects of PMA. Recent data suggest that bryostatin 1 and PMA affect certain isoenzymes of PKC in different ways (Szallasi *et al.*, 1994; Stanwell *et al.*, 1994). Twenty-four-hour exposure to PMA is associated with sustained activation of PKC $\alpha$ and $\beta$ in fibroblasts whereas bryostatin 1 induces significant down regulation of PKC $\alpha$ and $\beta$ (Isakow *et al.*, 1993). In view of this we anticipated that the PBMC taken from patients would have increased PKC activity in the first hour of treatment but that this would be significantly reduced by the end of the infusion.

To our knowledge, PKC has not been measured in humans before. These preliminary data show that the total and active PKC concentrations in the PBMCs of patients were modulated by bryostatin 1 treatment when compared with the PKC activity in the PBMCs of the normal woman who acted as a control. There were no consistent patterns of PKC activation between patients. In particular, there were no data to suggest down-regulation of total or active PKC by the end of the infusions. This may be due to the selective down-regulation of only some of the PKC isoenzymes by bryostatin 1, which would be undetectable by our assay system.

Two women with ovarian carcinoma responded to bryostatin 1. Both women had been treated with a platinum-containing drug regimen and then oral melphalan before receiving bryostatin 1. One woman had a partial response of 4 months' duration and the other had a minor response radiologically. Subsequently, both women developed progressive disease and the woman who had the minor response was treated with paclitaxel. Both are now taking tamoxifen and the first woman attained a partial response before developing progressive disease after 14 months' treatment, and the second woman has clinically stable disease after 10 months' treatment. This is interesting because tamoxifen is a powerful PKC inhibitor (Gescher, 1992), and therefore these women may have a subgroup of ovarian carcinoma which is sensitive to the manipulation of PKC. Whether bryostatin and tamoxifen modulate the activity of the same isoenzymes of PKC is not known.

*Immune function and cytokine studies*

Bryostatin 1 is immunomodulatory both *in vitro* and *in vivo*, but the immune effects in humans are unknown. LAK activity was of particular interest because LAK activity is associated with 25% response rates in patients with renal carcinoma or melanoma (Rosenberg *et al.*, 1987; Negrier *et al.*, 1989). In the three phase I trials of bryostatin 1, seven patients with renal carcinoma have been treated but none has responded to treatment. One partial and one minor response have been seen in melanoma (Philip, 1993), but the relevance of LAK activity in these patients is unknown. These data show that bryostatin 1 treatment is associated with a sustained increase in LAK-cell activity during the 4 week study period and a significant increase in the capacity of PBMCs to multiply in response to IL-2, although this declined over the 4 weeks of study. However, Figure 3 shows that the increase in LAK activity occurs during the first cycle of bryostatin 1. Whether subsequent cycles affect LAK activity is not clear from these data. It is possible that the decline in the proliferative response over 3 weeks is due to margination or extravasation of certain lymphocyte populations leading to an apparent reduction in the number or ability of cells to multiply in response to IL-2.

We have shown that bryostatin 1 inhibits LAK activity *in vitro* but increases LAK activity *in vivo* (Scheid *et al.*, 1994). This disparity led us to suspect that an indirect mechanism was responsible for the increased LAK activity *in vivo*. The mechanism by which bryostatin-1-stimulated $CD8^+$T cells reduced lung metastasis in a murine sarcoma model was shown to be mediated through the release of $\gamma$-interferon (Tuttle *et al.*, 1992). However, there was no measurable increase in the concentration of $\gamma$-interferon in the conditioned medium from patients' PBMCs (data not shown), and the mechanism for LAK cell stimulation in these patients remains unclear.

Philip *et al.* (1993) reported significant increases in the plasma concentration of TNF-$\alpha$ at 2 h and IL-6 at 24 h in 50% of the patients treated with $50\,\mu g\,m^{-2}$ per dose of bryostatin 1 over an hour. In order to investigate this, we measured the concentration of these cytokines in serum samples taken from 20 patients. Nine of the patients were from this trial (24 h infusion) and 11 were from the first phase I trial (1 h infusion), of whom three patients had received treatment at $65\,\mu g\,m^{-2}$ per dose. However, the serum samples from only one patient had raised levels of IL-6. These data suggest that the reported changes in cytokines are not present in all patients treated with high doses of bryostatin 1.

Bryostatin 1 is a novel anti-cancer agent that has shown clinical activity with this formulation and which is associated with modulation of protein kinase C activity and immune function *in vivo*. We recommend that bryostatin 1 is given as a 24 h infusion at a dose of $25\,\mu g\,m^{-2}$ per week for 8 weeks and that patients with ovarian carcinoma and low grade non-Hodgkin's lymphoma are entered into phase II trials.

**Acknowledgements**
The authors thank Sister V Goode, Sister A Watson and Mr R Caffrey for their assistance with this study. G Jayson, D Crowther, P Stern, A McGown and J Prendiville are funded by the Cancer Research Campaign. C Scheid is supported by the Deutsche Krebshilfe, Germany. G Pettit is grateful for the Outstanding Investigator Grant CA-44344-01A1-06 awarded by the division of Cancer Treatment, US National Cancer Institute, D.H.H.S.

**References**

AL-KATIB A, MOHAMMED RM, DAN M, HUSSEIN ME, AKHTAR A, PETTIT GR AND SENSENBRENNER LL. (1993). Bryostatin-1 induced hairy cell features on chronic lymphocytic leukaemia cells in vitro. *Exp. Haematol.*, **21**, 61–65.

BASU A, TEICHER BA AND LAZO JS. (1990). Involvement of protein kinase C in phorbol ester-induced sensitisation of HeLa cells to cisdiamminedichloroplatinum(II). *J. Biol. Chem.*, **265**, 8451–8457.

BENTLEY SA AND MILLER DT. (1986). Radionuclide blood cell survival studies. In *Methods in Haematology*, Vol. 14, *Radioisotopes in Haematology*, Lewis SM and Bayly RJ. (eds) pp. 245–262. Churchill Livingstone: Edinburgh.

BERKOW RL AND KRAFT AS. (1985). Bryostatin, a non-phorbol macrocyclic lactone, activates intact human polymorphonuclear leukocytes and binds to the phorbol ester receptor. *Biochem. Biophys. Res. Commun.*, **131**, 1109–1116.

BERKOW RL, SCHLABACH L, DODSON R, BENJAMIN WH, PETTIT GR, TUSTAGI P AND KRAFT AS. (1993). In vivo administration of the anticancer agent bryostatin-1 activates platelets and neutrophils and modulates protein kinase C activity. *Cancer Res.*, **53**, 2810–2815.

DALE IL AND GESCHER A. (1989). Effects of protein kinase C, including bryostatins 1 and 2 on the growth of A549 human lung carcinoma cells. *Int. J. Cancer*, **43**, 158–163.

DEKKER LV AND PARKER PJ. (1994). Protein kinase C – a question of specificity. *Trends Biochem. Sci.*, **218**, 73–77.

DELL'AQUILA ML, NGUYEN HT, HERALD CL, PETTIT GR AND BLUMBERG PM. (1987). Inhibition by bryostatin-1 of the phorbol ester induced blockage of differentiation in hexamethylene bisacetamide treated Friend erythroleukaemia cells. *Cancer Res.*, **47**, 6006–6009.

FIELDS AP, PETTIT GR AND MAY WS. (1988). Phosphorylation of laminin B at the nuclear membrane by activated protein kinase C. *J. Biol. Chem.*, **63**, 8253–8260.

GEBBIA V, CITARELLA P, MISERENDINO V, VALENZA R, BORSELLINO N, PESTA A, PETTIT R AND MAY S. (1992). The effects of the macrocyclic lactone bryostatin-1 on leukaemic cells in vitro. *Tumori*, **78**, 167–171.

GESCHER A. (1985). Antiproliferative properties of phorbol ester tumour promoters. *Biochem. Pharmacol.*, **34**, 2587–2592.

GESCHER A. (1992). Towards selective pharmacological modulation of protein kinase C-opportunities for the development of novel anti-neoplastic agents. *Br. J. Cancer*, **66**, 10–19.

GIGNAC SM, BUSCHLE M, PETTIT GR, HOFFBRAND AV AND DREXLER HG. (1990). Expression of proto-oncogene c-jun during differentiation of B-chronic lymphocytic leukaemia. *Leukaemia*, **4**, 441–444.

HENNINGS H, BLUMBERG PM, PETTIT GR, HERALD CL, SHORES R AND YUSPA S. (1987). Bryostatin-1, an activator of protein kinase C, inhibits tumour promotion by phorbol esters in Sencar mouse skin. *Carcinogenesis*, **9**, 1343–1346.

HICKMAN PF, WADE K, KEMP GJ, HARRIS AL, TAYLOR DJ, STYLES P AND RADDA GK. (1993). Preliminary investigation of bryostatin induced myalgia using 31P magnetic resonance spectroscopy. Proceedings of the Society for Magnetic Resonance in Medicine, 12th annual scientific meeting, New York, p. 1130.

HORNUNG RL, PEARSON JW, BECKWITH M AND LONGO DL. (1992). Preclinical evaluation of bryostatin as an anticancer agent against several murine tumour cell lines: in vitro versus in vivo activity.*Cancer Res.*, **52**, 101–107.

ISAKOW N, GALRON D, MUSTELIN T, PETTIT GR AND ALTMAN A. (1993). Inhibition of phorbol ester induced T cell proliferation by byrostatin is associated with rapid degradation of protein kinase C. *J. Immunol.*, **150**, 1195–1204.

JONES RJ, SHARKIS SJ, MILLER CB, ROWINSKY EK, BURKE PJ AND MAY WS. (1990). Bryostatin-1, a unique biologic response modifier: anti-leukaemic activity in vitro. *Blood*, **75**, 1319–1323.

KENNEDY MJ, PRESTIGIACOMA LJ, TYLER G, MAY WS AND DAVIDSON NE. (1992). Differential effects of bryostatin 1 and phorbol ester on human breast cancer cell lines. *Cancer Res.*, **52**, 1278–1283.

KISS Z, DELI E AND GIRARD PR. (1987). Comparative effects of polymixin B, phorbol ester and bryostatin on protein phosphorylation, protein kinase C translocation, phospholipid metabolism and differentiation of HL 60 cells. *Biochem. Biophys. Res. Commun.*, **146**, 208–215.

KRAFT AS, SMITH JB AND BERKOW RL. (1986). Bryostatin, an activator of the calcium phospholipid dependent protein kinase, blocks phorbol ester induced differentiation on human promyelocytic leukaemia cells HL-60. *Proc. Natl Acad. Sci. USA*, **83**, 1334–1338.

KRAFT AS, BAKER VV AND MAY WS. (1987). Bryostatin induces changes in protein kinase C location without altering c-myc expression in human promyelocytic leukaemia cells (HL60). *Oncogene*, **1**, 111–118.

KRAFT AS, WILLIAM F AND PETTIT GR. (1989). Varied differentiation responses of human leukaemias to bryostatin-1. *Cancer Res.*, **49**, 1287–1293.

LAMB M. (1992). A peroxidase linked enzyme immunoassay for TNF-α utilising alternative colorimetric or chemilumimetric substrates. *J. Immunol Methods*, **155**, 215–223.

LEONARD JP, MAY WS, IHLE JN, PETTIT GR AND SHARKIS SJ. (1988). Regulation of haematopoiesis-IV: the role of interleukin 3 and bryostatin-1 in the growth of erythropoietic progenitors from normal and anaemic W/W$^v$ mice. *Blood*, **72**, 1492–1496.

MCCRADY CW, STANISWALIS J, PETTIT GR, HOWE C AND GRANT S. (1991). Effects of pharmacologic manipulation of protein kinase C by phorbol dibutyrate and bryostatin-1 on the clonogenic response of human granulocyte macrophage progenitors to recombinant GM-CSF. *Br. J. Haematol.*, **77**, 5–15.

MAY WS, SHARKIS SJ, ESA AH, GEBBIA U, KRAFT AS, PETTIT GR AND SENSENBRENNER L. (1987). Antineoplastic bryostatins are multipotential stimulators of human haematopoietic progenitor cells. *Proc. Natl Acad. Sci. USA*, **84**, 8483–8487.

MOHAMMAD RM, AL-KATIB A, PETTIT GR AND SENSENBRENNER LL. (1993). Differential effects of bryostatin-1 on human non-Hodgkins lymphoma cell lines. *Leuk. Res.*, **17**, 1–8.

MOHAMMED RM, AL-KATIB A, PETTIT GR AND SENSENBRENNER LL. (1994). Successful treatment of human Waldenstrom's macroglobulinaemia with combination biological and chemotherapy agents. *Cancer Res.*, **54**, 165–168.

NEGRIER S, PHILLIP T AND STOTER G., *et al.* (1989). Interleukin 2 with or without LAK cells in metastatic renal carcinoma: a report of a European multicentre study. *Eur. J. Cancer Clin. Oncol.*, **25**, S3 S31–S28.

NUTT JE, HARRIS AL AND LUNEC J. (1991). Phorbol ester and bryostatin effects on growth and the expression of oestrogen responsive and TGF-b1 genes in breast tumour cells. *Br. J. Cancer*, **64**, 671–676.

PETTIT GR, HERALD CL AND DOUBEK DL. (1982). Isolation and structure of bryostatin-1. *J. Am. Chem. Soc.*, **104**, 6846–6848.

PHILIP PA, REA D, THAVASU P, CARMICHAEL J, STUART NSA, ROCKETT H, TALBOT DC, GANESAN T, PETTIT GR, BALKWILL F AND HARRIS AL. (1993). Phase I study of bryostatin-1: assessment of interleukin-6 and tumour necrosis factor a in vivo. *J. Natl Cancer Inst.*, **85**, 1812–1818.

PRENDIVILLE J, CROWTHER D, THATCHER N, WOLL PJ, FOX BW, MCGOWN A, TESTA N, STERN P, MCDERMOTT R, POTTER M AND PETTIT GR. (1993). A phase I study of intravenous bryostatin-1 in patients with advanced cancer. *Br. J. Cancer*, **68**, 418–424.

ROSENBERG SA, LOTZE MT, MUUL LM, CHANG AE, AVIS FP, LEITMAN S, LINEHAN WM, ROBERTSON CN, LEE RE, RUBIN JT, SEIPP CA AND SIMPSON CGA. (1987). A progress report on the treatment of 157 patients with advanced cancer using lymphokine activated killer cells and interleukin 2 or high dose interleukin 2 alone. *N. Engl. J. Med.*, **316**, 889–897.

SCHEID C, PRENDIVILLE J, JAYSON GC, CROWTHER D, FOX B, PETTIT GR AND STERN PL. (1994). Immunomodulation in patients receiving intravenous bryostatin-1 in a phase I clinical study: comparison with effects of bryostatin-1 on lymphocyte function in vitro. *Cancer Immunol. Immunother.* (in press).

SCHUCHTER LM, ESA AH, MAY WS, LAULIS MK, PETTIT GR AND HESS AD. (1991). Successful treatment of murine melanoma with bryostatin 1. *Cancer Res.*, **51**, 682–687.

SHARKIS SJ, JONES RJ, BELLIS ML, DEMITRI GD, GRIFFIN JD, CIVIN C AND MAY WS. (1990). The action of bryostatin on normal human haematopoietic progenitors is mediated by accessory cell release of growth factors. *Blood*, **76**, 716–720.

STANWELL C, GESCHER A, BRADSHAW T AND PETTIT GR. (1994). The role of protein kinase C isoenzymes in the growth inhibition caused by bryostatin-1 in human A549 lung and MCF-7 breast carcinoma cells. *Int. J. Cancer*, **56**, 585–592.

STONE RM, SARIBAN E, PETTIT GR AND KUFE DW. (1988). Bryostatin-1 activates protein kinase C and induces monocytic differentiation of HL-60 cells. *Blood*, **72**, 208–213.

SZALLASI Z, SMITH CB, PETTIT GR AND BLUMBERG PM. (1994). Differential regulation of protein kinase C isoenzymes and phorbol 12-myristate 13-acetate in NIH 3T3 fibroblasts. *J. Biol. Chem.*, **269**, 2118–2124.

TUTTLE TM, INGE TH, BETHKE KP, MCCRADY CW, PETTIT GR AND BEAR HD. (1992). Activation and growth of murine tumour specific T cells which have in vivo activity with bryostatin-1. *Cancer Res.*, **52**, 548–553.

WARREN BS, KAMANO Y, PETTIT GR AND BLUMBERG PM. (1988). Mimicry of bryostatin-1 induced phosphorylation patterns in HL-60 cells by high phorbol ester concentrations. *Cancer Res.*, **48**, 5984–5988.

WHETTON AD, HEYWORTH CM, NICHOLLS SE, EVANS CA, LORD JM, DEXTER TM AND OWEN-LYNCH PJ. (1994). Cytokine mediated protein kinase C activation is a signal for lineage determination in bipotential granulocyte macrophage colony forming cells. *J. Cell Biol.*, **125**, 651–659.

WHO (1979). *Handbook for reporting Results of Cancer Treatment.* World Health Organization: Geneva.

WILLIAM F, MROCZKOWSKI B, COHEN S AND KRAFT AS. (1988). Differentiation of HL60 cells with an increase in the 35 kD protein lipocortin 1. *J. Cell Physiol.*, **137**, 402–410.